# Supplementary material for: Are carbon emissions trading and green financial instruments synergistic? -Comprehensive quantitative research based on content analysis
Source: PLoS One. 2024 Mar 7;19(3):e0298601. doi: 10.1371/journal.pone.0298601 (PMC10919601; doi:10.1371/journal.pone.0298601)
Supplement: S1 Data — (ZIP) [file pone.0298601.s001.zip › Data/地级市Z_F工作报告.zip!/╣π╢1⁄2/╜╥╤⌠╩╨/└┤╫╘╣1⁄2╓┌║┼í╛┬φ┐╦╩2╛▌═°í┐_2009.docx]

各位代表：

　　我代表市人民政府向大会作政府工作报告，请予审议，并请政协各位委员和其他列席人员提出意见。

　　一、2008年工作回顾

　　2008年，在省委、省政府和市委的正确领导下，在市人大及其常委会和市政协的监督支持下，市政府坚持以科学发展观为统领，团结带领全市人民，开拓进取，奋力拼搏，在攻坚克难中取得了新成绩，在推进科学发展中实现了新跨越。市四届人大三次会议确定的目标任务基本完成。

　　（一）经济保持较快发展

　　经济步入较快健康发展轨道，主要经济指标增速名列全省前茅。全市生产总值725.03亿元，比上年（下同）增长16%；地方财政一般预算收入22.37亿元，增长29.13%，其中税收收入17.11亿元，增长31.95%；全社会固定资产投资265.79亿元，增长32%；规模以上工业增加值214.73亿元，增长36.7%；社会消费品零售总额265.29亿元，增长27%；外贸出口总额20.98亿美元，增长18.6%；实际利用外资2.04亿美元，增长34%。经济质量和效益有所增强，财税收入结构逐步优化。新认定中国高新技术企业24家，新增规模以上工业企业362家、省级民营科技企业12家。珠宝玉器质量监督检验站（揭阳）通过认证，国家不锈钢制品质量监督检验中心获批筹建。珠海（揭阳）产业转移工业园获省批准设立，省市“共建支柱产业”步伐加快。经贸交流活动取得新成效。金融支持力度不断加大，贷款余额比年初增长13.7%；首批银企45亿元融资协议已获授信额度30.8亿元。

　　（二）重点项目有新突破

　　揭阳潮汕机场征地拆迁、移民安置等工作圆满完成，厦深铁路揭阳段征地拆迁工作全面铺开。惠来电厂3、4号机组及码头扩建工程扎实推进；乌屿大型核电项目初可研通过专家组评审；中石油、中海油、中电投、华润、国电等重大项目正抓紧跟踪落实。汕揭、潮揭高速公路揭阳段加快推进；国道206线、省道236线、省道335线有关路段大修工程进展顺利。粤东首个LNG卫星站建成投入运营。500千伏榕江、220千伏紫峰、铁山输变电工程和惠来电厂1、2号机组送电线路建成投产。三洲拦河闸等重点水利工程抓紧建设，韩江引水工程列入省规划。市区新老“30亿工程”扎实推进，一批市政设施建成投入使用，市区及各县（市）城区功能逐步配套，城市面貌明显改观。

　　（三）“三农”工作得到加强

　　农村经济稳步发展，总收入478.1亿元，增长5.9%。强农惠农政策有效落实，扶贫开发投入7800万元，完成通村公路硬底化400公里，农村生产生活条件进一步改善。农业产业化水平不断提高，新增全国农业龙头企业1家、省重点农业龙头企业2家、省级农业名牌产品5个、省级现代农业园区2个、农民专业合作社46个。农业生产获得丰收，粮食播种面积209.2万亩，总产87.3万吨，分别增长2.6%和6.1%。宜林荒山造林计划稳步实施，森林覆盖率51.2%。农村信息化综合信息服务试点工作全面铺开。玉湖经验得到推广落实，村（居）委会换届选举工作全面完成，新农村建设取得新成效。

　　（四）人民生活逐步改善

　　就业工作扎实推进，新增就业3.01万人，农村劳动力转移就业8.96万人，下岗失业人员实现再就业1.2万人，城镇登记失业率3.07%。社会保障不断加强，参加企业养老保险18.9万人，征缴收入3.7亿元，支付养老待遇3.66亿元；新型农村合作医疗参合率97.28%，参加城镇居民和职工医疗保险52.17万人；城乡居民享受低保待遇12.42万人，实现应保尽保。城乡中小学生义务教育免收学费4.86亿元。城镇居民人均可支配收入11757元，增长9.4%；农村居民人均纯收入4926元，增长8%。

　　（五）社会事业加快发展

　　筹措教育建设资金24.24亿元，新建扩建校舍210万平方米，增配教师5115名，在校高中生增加1.74万人。高考取得好成绩。公共卫生服务体系进一步完善，乡村卫生站实现全覆盖。阳美翡翠玉雕等5个项目入选第二批国家级非物质文化遗产名录。成功举办首届特色文化节。首获省“双拥模范城”称号。完成《揭阳市环境保护规划》和《揭阳市水环境综合整治实施方案》编制工作，节能减排取得新成效。违法违规用地行为得到有效整治，市级地质灾害防治规划完成编制。人口计生工作不断加强，人口出生率11.33‰，自然增长率6.28‰。妥善应对处置“三鹿奶粉事件”，产品质量和食品药品安全得到有效监管。安全生产形势保持稳定。集中开展十大整治专项行动，刑事发案比上年减少14.8%。信访工作不断加强，社会保持稳定和谐。抗震救灾工作扎实开展。华侨农场改革改制稳步实施。其他各项工作取得新进展。

（六）民主法治扎实推进

　　认真执行市人大及其常委会的决议决定，加强与人民政协的沟通，自觉接受监督。办理人大代表议案、建议51件，政协委员提案109件。重视工会、共青团、妇联等人民团体和市民群众意见，依法决策、科学决策、民主决策机制进一步完善。依法行政工作扎实推进。行政审批制度改革不断深化，政务公开工作取得新成效。廉政建设不断加强，机关作风效能建设深入开展，市、县行政电子监察系统全面建成。经济责任审计进一步强化，一批违法违纪案件受到查处。

　　各位代表，过去一年，我们经历了种种困难，战胜了各种挑战，成绩来之不易。这是省委、省政府和市委正确领导的结果，是全市人民团结奋斗、各界朋友大力支持的结果。在此，我谨代表市人民政府，向全市广大工人、农民、知识分子、干部职工，向驻揭部队、武警官兵、人民警察和各民主党派、人民团体、各界人士，向关心支持我市发展的港澳台同胞、海外侨胞、外出乡亲，表示衷心的感谢并致以崇高的敬意！

　　我们也清醒地看到，我市经济社会发展仍存在不少困难和问题：受国际金融危机、物价上涨等因素影响，生产总值虽完成绝对值，但实际增长率比预期低3个百分点；经济发展质量不高，人均水平差距明显；发展方式粗放，节能减排任务依然繁重；教育、卫生、文化等社会事业发展仍较滞后，城乡居民收入水平有待提高；社会治安形势仍不容乐观，维稳压力依然较大。对此，我们将以对人民高度负责的态度，采取更加有效的措施，在加快发展中认真解决。

　　二、2009年工作基本思路和目标

　　2009年是我市扎实推进科学发展，坚持改革创新，实现后来居上的重要一年。当前，金融危机仍未见底，经济下行压力巨大。我们既要看到这次危机带来的不利影响，宁可把困难估计得更严重一些，把应对措施准备得更充分一些，同时更要看到，我国仍处在重要战略机遇期，经济发展的基本面和长期向好的趋势没有改变。特别是去年以来，中央和省出台了一系列扩内需、保增长、调结构的政策措施，为我市加快重点项目建设、调整优化产业结构带来了难得的发展机遇。我们一定要咬定工作目标不放松，坚定发展信心不动摇，抢抓历史机遇不懈怠，变压力为动力，化挑战为机遇，坚定不移地推进揭阳科学发展。

　　今年政府工作的基本思路是：以邓小平理论和“三个代表”重要思想为指导，以科学发展观统领经济社会发展全局，认真贯彻党的十七大、十七届三中全会、中央经济工作会议、省委十届四次全会和市委四届六次全会精神，全面实施“一三二”发展思路，把“三加快一确保”作为工作主线，坚持解放思想和改革创新，坚持扩大投资和拉动消费，坚持调整结构和增强效益，坚持节约资源和生态保护，坚持保障民安和改善民生，全力以赴推动经济社会更好更快发展。

　　主要预期目标是：生产总值增长11%，单位生产总值能耗下降3%，二氧化硫和化学需氧量分别削减6.25%和2.52%，规模以上工业增加值增长21.5%，全社会固定资产投资增长30%以上，社会消费品零售总额增长28%，外贸出口增长8%，实际利用外资增长20%，地方财政一般预算收入增长20%以上，居民消费价格总水平涨幅控制在4%以内，城镇登记失业率控制在3.5%以内，城镇居民人均可支配收入增长12%，农民人均纯收入增长10%，人口自然增长率控制在7.4‰以内。

　　三、全力确保经济持续较快增长

　　充分调动一切积极因素，沉着应对金融危机影响，抓住国家实施积极财政政策和适度宽松货币政策的重大契机，千方百计扩大投资、拉动消费，促进提高自主创新能力，促进传统产业优化升级，促进建设现代产业体系，确保经济持续较快增长。

　　（一）以扩大投资规模为支撑，加快基础设施和重大项目建设

　　加快完善交通环境。全力支持和推进揭阳潮汕机场及场外配套工程建设，完成厦深铁路揭阳段征地拆迁任务。加快高速公路规划建设，力促汕揭高速公路早日建成通车；抓紧做好省网二横和省网四横揭阳段、揭惠（揭阳潮汕机场至惠来神泉港）等高速公路前期工作。加快国道206线曲溪至小坑段、省道335线深坑至曲溪段和机场进场路建设，尽快开工国道324线揭阳段、省道335线霖磐至龙尾段改造工程，确保国道206线曲丰段、省道236线池揭段大修和陈沙公路延长线按期建成通车，加快莲花大桥及连接线的规划建设，使全市交通环境明显改善。

加快推进重大项目。提高惠来电厂1、2号机组发电效益，争取3、4号机组项目尽快核准建设。加强跟踪落实，力争惠来乌屿核电厂和中石油、中海油等央企重大合作项目顺利落户。加快仙安风电场建设，推进华润、国电、中电投等电力能源项目前期工作。争取开工220千伏桑浦、兰花、映月、沟美输变电工程等项目。做好全市电网规划建设，完善电网结构，满足送电供电需要。

　　加快市政配套建设。按照打造岭南水城和特色城市群的要求，尽快完成《揭阳市城市总体规划》修编工作。全力推进市区新老“30亿工程”，完成环市北路、晓翠北路、海关至南河大桥段等市政工程，启动莲花大道北段、梅兜路等新“30亿工程”第二批项目，加快临江北路绿化景观带配套建设。争取国家尽快核准榕江大围城市防洪工程。继续落实各县（市）每年筹资1亿元投入市政设施建设，尽快形成特色城市群。

　　（二）以自主创新为动力，调整优化产业结构

　　扶强做大现有工业企业。坚定不移地推进工业发展“三个新增100”。落实各项政策措施，完善领导干部挂钩扶持重点企业制度，鼓励和引导企业增资扩产，加强技术改造，力促中小企业发展壮大。抓好企业股份制改造工作，鼓励和支持高成长性企业上市融资，提高资本运营水平。

　　增强企业自主创新能力。积极实施科技计划项目，引导创新要素向企业集聚。鼓励企业设立技术研发机构，加快推进产学研结合，逐步建立起以企业为主体的技术创新体系。支持企业争创自主品牌，大力发展高技术含量、高附加值产品，促进科技成果转化，推动产业优化升级。加强以科技园、产业基地和专业镇为主体的科技孵化基地建设，为科技型企业发展提供良好载体。加快国家不锈钢制品质量监督检验中心建设，树立行业权威，提高行业市场竞争力。强化知识产权保护，做好技术标准推广工作。

　　推动产业集聚发展。加强本土企业间的经济技术合作，围绕龙头企业发展配套工业，开发下游产品，拉长产业链和产品链，形成产业集群。扎实推进“双转移”工作，主动承接产业转移，引导企业向园区集聚。加快珠海（揭阳）产业转移园区基础设施建设，尽快形成投资环境，争创省级产业转移示范园。高起点规划建设大南海国际石化综合工业园，抓紧推进空港经济区等园区规划建设，为加快产业集聚发展拓展空间。

　　加快建设现代产业体系。巩固发展劳动密集型、轻型加工业，运用高新技术和先进适用技术改造提升五金机械、纺织服装、化工塑料、食品制药等传统优势产业，做大做强支柱产业。调整发展重心，编制实施沿海重化产业带发展规划，重点发展电力能源、石油化工、装备制造等重化产业；加快发展现代服务业，编制实施大型专业市场和现代物流发展规划，重点建设药品、玉器、轻纺、数码、金属材料和再生资料等现代服务基地，逐步提高第三产业比重，打造具有揭阳特色的现代产业体系。

　　（三）以扩大消费需求为着力点，全面搞活内外经贸

　　积极开拓国内消费市场。坚持“走出去”与“请进来”相结合，组织企业参加各类经贸活动，鼓励和帮助加工贸易企业扩大产品内销，引导企业积极开拓省外市场。继续办好玉器节、衬衣节、五金不锈钢制品博览会等特色节会，进一步搞旺商贸流通，提高揭货的市场占有率。

　　千方百计扩大对外贸易。巩固传统外贸市场，大力拓展俄罗斯、中东、东欧、非洲、南美等新兴市场。深入实施科技兴贸和品牌带动战略，优化提升一般贸易出口结构，推进加工贸易企业转型升级。积极引进先进技术，扩大急需原材料进口。做好扶持和服务外贸企业工作，加强加工贸易联网监管和电子口岸建设，争取获批一类口岸，优化大通关环境，提高通关效能。

　　提高利用内外资水平。建立健全以县（市、区）为主体的招商机制，推动招商工作重心下移。加强对签约项目的跟踪服务，提高项目履约落地率。充分发挥侨资侨力丰富优势，增强利用外资工作成效。继续实施“乡贤回归”工程，积极动员在外揭籍企业家回乡投资创业。把招商引资重点瞄准中央及省属国有企业，争取引进一批大型项目落户。

加强区域交流与协作。积极参与粤东四市经济社会协作，扩大闽粤赣十三市区域交流合作，密切与珠三角、海峡西岸经济区的联系，大力拓展区域发展空间。

　　（四）以促进农业稳定发展、农民持续增收为目标，加强农业农村工作

　　增加对农业农村投入。认真落实种粮补贴、生猪补贴、农机购置补贴等强农惠农政策，提高农村金融服务能力，促进农业生产稳定发展。加快现代标准农田建设，实施沃土工程，全年力争改造低产田6万亩以上。加快三洲拦河闸等重点水利设施建设，尽快启动韩江引水工程，抓好水库、堤围除险加固，提高水利灌溉和防灾减灾能力。大力推进农村饮水工程建设，切实解决群众饮水安全问题。落实扶持项目和资金，加快扶贫开发步伐。

　　加快农业产业化进程。稳定和完善农村基本经营制度，推进集体林权制度改革，规范土地承包经营权流转，促进适度规模经营。大力发展现代农业，增强农业龙头企业带动作用，促进农民增收致富。加强农产品市场建设，健全农民专业合作经济组织和农业生产综合服务体系，深入开展农村信息化综合信息服务试点工作。依托各地资源优势，大力发展特色优势产业和产品，打造一批专业镇、专业村。

　　推进城乡一体化。深化农村各项改革，扎实推进“镇改街”、“村改居”工作。继续推广玉湖经验，加快新农村建设步伐。认真搞好农村村庄建设规划修编，抓紧推进旧村庄改造，强化农村环境综合整治，建设生态和谐文明村。统筹城乡基础设施建设和公共服务，抓好12个中心镇的规划建设，增强城镇辐射带动农村能力。

　　四、努力促进社会稳定和谐

　　越是经济困难的时候，越要高度重视民生保障，大力加强社会建设，确保社会稳定和谐。

　　（一）统筹社会事业发展，提高公共服务水平

　　办好人民满意教育。千方百计筹措建设资金，加快中小学校舍建设，提供更多优质学位。全力推进10所高（职）中建设，确保今年秋季建成投入使用。整合教育资源，优化学校布局，促进义务教育均衡发展。做好学生防流控辍工作，推行中职学校春季招生。加强师资队伍建设，落实中小学教师工资福利“两相当”工作，逐步提高教师福利待遇水平，尽快解决代课教师问题。鼓励、支持和规范民间办学，大力发展职业教育和技工教育。切实加强揭阳职业技术学院、潮汕学院建设。

　　丰富城乡文化生活。抓紧推进揭阳楼、学宫修缮、学宫广场、市文化中心、体育馆等项目建设，配套完善县、镇、村文化设施，活跃群众文化生活。加强文物、非物质文化遗产的保护、管理和合理利用工作。积极发展社会科学、文学艺术、新闻出版、广播电视和体育等事业。

　　协调推进各项事业。加强疾病预防控制和卫生监督，加快基层医疗设施建设，提高医疗服务质量和防病治病水平。扎实推进中医药大市建设。扩大新农合覆盖面。开工建设市卫生学校新校区，早日建成市人民医院新住院大楼。落实优抚安置政策，巩固双拥工作成果。积极发展老龄、妇幼和残疾人事业。继续抓好华侨农场改革改制工作。重视做好旅游、外事、侨务、对台、宗教、人防、气象、地震、档案、史志等工作。

　　（二）加强各项管理工作，提高城市综合竞争力

　　打造诚信揭阳。继续严厉打击走私贩私、制假售假、偷税逃税等违法犯罪行为，加强物价监管，查处取缔无证照经营行为，强化税收征管和税务稽查，进一步规范市场秩序，营造公平竞争的良好环境。完善工商、税务、财政、海关、银行、质监、药监、统计等经济管理部门信息共享制度，加强信用体系建设，改善和优化金融生态环境。完善金融工作联席会议制度，进一步密切政银企关系；建立健全小企业授信新机制，构建多层次信用担保体系，争取金融机构加大对地方重点项目和中小企业的信贷支持。

　　打造生态揭阳。加强环境保护，促进经济社会可持续发展。加快推进19座污水处理厂和6个垃圾填埋场建设，确保列入省计划的9座污水处理厂年底前全部建成投入使用。全面实施《揭阳市水环境综合整治方案》，突出抓好榕江、练江流域整治，加大水资源保护力度。强化海洋国土观念，合理开发、利用海洋资源。继续抓好违法违规用地整治工作，加强土地矿产资源利用规划和节约集约管理，提高综合利用水平。强化督查考核，严格控制人口增长，提高人口素质。完善殡葬设施，巩固殡改成果。构筑生态公益林新体系，建设绿色揭阳。

　打造文明揭阳。深入开展群众性精神文明创建活动，争创省级文明城市和卫生城市。加强公民思想道德建设，提高市民文明素质。倡导移风易俗，培养良好社会风尚。弘扬潮汕优秀文化，打造“自强自信、创业创新、和谐开放、感恩奉献”的特色文化。整顿市区交通营运秩序，规范小区物业管理。强化城市管理行政执法，全面落实“门前三包”责任制，大力整治“脏、乱、差”现象，创造优美整洁城市环境。

　　打造和谐揭阳。建立健全应急管理体制机制，有效应对自然灾害、事故灾难、公共卫生和社会安全突发事件。强化产品质量管理，确保食品药品安全。加强安全生产，严防重特大安全生产事故发生，保障人民群众生命财产安全。健全信访工作机制，畅通信访渠道，规范信访行为，及时、妥善解决群众合理诉求。加强司法援助和法律援助工作，保护弱势群体合法权益。加强情报信息工作，及时有效化解不稳定因素。继续深入开展社会治安源头治理行动，加快群防群治和治安防控体系建设，完善社会治安视频监控系统，提高治安动态监控能力，维护社会稳定。

　　（三）保障和改善民生，提高人民群众生活水平

　　积极促进和扩大就业。加强就业公共服务，完善人力资源市场信息网络和服务体系，拓宽劳动者就业渠道，促进就业和再就业，确保城镇新增就业3.1万人。落实积极就业政策，以创业带动就业。认真做好返乡农民工工作，加强劳动力技能培训和转移输出。抓好大中专毕业生就业工作，完善城乡就业援助制度。

　　强化民生保障。调整优化财政支出结构，加大民生投入。认真做好医疗保险合并管理试点工作，妥善解决早期离开国有集体企业人员社会保险、困难企业退休人员参加基本医疗保险和被征地农民养老保险等问题。抓好社保扩面征缴工作，加强基金支出管理，确保社保待遇按时足额发放。全面落实城乡居民最低生活保障制度，保障困难群众基本生活。

　　加大扶危济困力度。大力倡导扶危济困社会风尚，发展社会慈善福利事业，积极做好救济救助工作。抓紧推进市区廉租房第一期项目建设，切实解决城镇低收入家庭住房困难。落实援建资金，做好对口支援汶川卧龙灾区重建工作。

　　五、大力加强政府自身建设

　　按照“为民、务实、清廉”的要求，加强政府自身建设，优化政务环境，提高工作效能。

　　（一）坚持执政为民，建设服务政府

　　深入开展学习实践科学发展观活动，切实转变机关作风。继续抓好行政审批制度改革，清理和取消不必要的前置审批，推行限时办结制、服务承诺制、重要岗位AB角制等办事制度。加强电子政务建设，扩大网上审批范围，为企业提供优质服务。发挥电子监察系统作用，加强对行政审批的在线监督、实时监控。严格控制针对企业的检查评比活动，多为企业排忧解难，切实减轻企业负担。

　　（二）坚持依法行政，建设法治政府

　　贯彻落实《国务院关于加强市县政府依法行政的决定》，严格依法行使权力，规范行政执法行为。完善行政决策机制，进一步提高依法、科学、民主决策水平。加强政府法制工作，强化行政执法监督和行政复议。加大政务公开力度。加强严格执法和法制宣传，推进依法治市进程。认真执行市人大及其常委会的决议决定，支持市政协履行职能，及时办理人大代表议案、建议和政协提案，认真听取民主党派、工商联、无党派人士和人民团体的意见建议，自觉接受各方面监督。

　　（三）坚持行政问责，建设责任政府

　　贯彻执行《广东省各级政府部门行政首长问责暂行办法》和《揭阳市影响机关效能行为责任追究办法》等规定，严格落实各项责任制度，实行行政问责制，对失职、渎职、不作为等进行问责追究。加强督促检查，狠抓决策部署的落实。完善绩效考核机制，改进绩效考评办法，坚决兑现奖罚，提高政府执行力。

　　（四）坚持从严治政，建设廉洁政府

抓好廉政建设和反腐败工作，营造风清气正的干事环境。发扬艰苦奋斗作风，树立过紧日子思想，勤俭办一切事业。加强行政监察和审计监督，确保公务购车和用车经费、会议经费、公务接待费用、党政机关出国（境）费用、办公经费预算实现“五个零增长”，由财政出资或党政机关主办的晚会、展览、庆典、论坛活动实现“四个减半”。

　　各位代表！做好今年各项工作，实现全年预期目标，任务艰巨，使命光荣，责任重大。让我们紧密团结在以胡锦涛同志为总书记的党中央周围，在省委、省政府和市委坚强领导下，同心同德，振奋精神，开拓创新，扎实工作，为建设富民强市、和谐文明新揭阳而努力奋斗！
